# Supplementary material for: Stacking Model‐Based Classifiers for Dealing With Multiple Sets of Noisy Labels
Source: Biom J. 2025 Mar 12;67(2):e70042. doi: 10.1002/bimj.70042 (PMC11898607; doi:10.1002/bimj.70042)
Supplement: Supplementary file 1 — Supporting Information [file BIMJ-67-e70042-s001.zip › density-based-ensemble-model-main/competitors_real_data/netlab-master/netlab-master/help/minbrack.htm]

Netlab Reference Manual minbrack


# minbrack

## Purpose

Bracket a minimum of a function of one variable.

## Description

`brmin, brmid, brmax, numevals] = minbrack(f, a, b, fa)`
finds a bracket of three points around a local minimum of
`f`. The function `f` must have a one dimensional domain.
`a < b` is an initial guess at the minimum and maximum points
of a bracket, but `minbrack` will search outside this interval if
necessary. The bracket consists of three points (in increasing order)
such that `f(brmid) < f(brmin)` and `f(brmid) < f(brmax)`.
`fa` is the value of the function at `a`: it is included to
avoid unnecessary function evaluations in the optimization routines.
The return value `numevals` is the number of function evaluations
in `minbrack`.

`minbrack(f, a, b, fa, p1, p2, ...)` allows additional
arguments to be passed to `f`

## Examples

An example of the use of this function to bracket the minimum of a function
`f` in the direction `sd` can be found in `linemin`

```
[min, mid, max, nevals]] = minbrack('linef', 0.0, 1.0, fa, f, pt, dir);
```

where the function `linef` is used to turn a general function `f`
into a one dimensional one.

## Algorithm

Quadratic extrapolation with a limit to the maximum step size is
used to find the outside points of the bracket. This implementation
is based on that in Numerical Recipes.

## See Also

`linemin`, `linef`

---

**Pages:**
Index


---

Copyright (c) Ian T Nabney (1996-9)
